# Defining associated factors for total fertilization failure and embryo development arrest in intracytoplasmic sperm injection cycles

**DOI:** 10.1186/s12905-026-04384-4

**Published:** 2026-03-09

**Authors:** Mustafa Akşar, Serdar Dilbaz

**Affiliations:** University of Health Sciences, Etlik Zubeyde Hanim Women’s Health Training and Research Hospital, Ankara, Turkey

**Keywords:** Intracytoplasmic sperm injection (ICSI), Total fertilization failure, Embryo developmental arrest

## Abstract

**Background:**

Total fertilization failure (TFF) and embryo developmental arrest (EDA) remain clinically challenging outcomes in intracytoplasmic sperm injection (ICSI) cycles. Although ICSI is widely and effectively used to overcome fertilization disorders, fertilization failure and embryo development arrest continue to occur in a subset of patients. This study aimed to identify baseline clinical characteristics and ovarian reserve parameters associated with the risk of TFF and EDA.

**Methods:**

In this retrospective cohort study, 1,846 ICSI cycles performed between January 2016 and December 2019 at a tertiary assisted reproduction center were analyzed. Cycles were categorized as successful fertilization, total fertilization failure (TFF), or embryo developmental arrest (EDA). For regression analyses, TFF and EDA were combined as unsuccessful fertilization. Baseline pre-treatment variables, including female age, basal follicle-stimulating hormone (FSH), serum anti-Müllerian hormone (AMH), antral follicle count, infertility etiology, and semen characteristics, were evaluated. Univariate and multivariable logistic regression analyses were performed, and internal validation was conducted using bootstrap resampling.

**Results:**

Successful fertilization occurred in 76.5% of cycles, whereas TFF and EDA were observed in 6.6% and 16.9%, respectively. In univariate analyses, female age, basal FSH, serum AMH, antral follicle count, estradiol level on the day of oocyte pick-up, and oocyte yield parameters were significantly associated with fertilization outcomes. In the multivariable model restricted to baseline variables, female age (adjusted OR 0.94, 95% CI 0.91–0.97), basal FSH (adjusted OR 0.93, 95% CI 0.89–0.97), serum AMH (log-transformed; adjusted OR 1.42, 95% CI 1.18–1.72), and infertility etiology remained independently associated with fertilization success. The optimism-corrected AUC was 0.63.

**Conclusions:**

Diminished ovarian reserve markers were independently associated with increased risk of TFF and EDA. Although statistically significant, the predictive performance was modest, supporting use in pre-treatment counseling rather than definitive prediction.

## Introduction

Intracytoplasmic sperm injection (ICSI) is an effective method applied to increase fertilization rates in cycles that do not fertilize or have low fertilization rates after conventional in vitro fertilization (IVF) treatment [1]. Current evidence identifies Intracytoplasmic Sperm Injection (ICSI) as the most established and clinically effective intervention for couples with severe male factor infertility. Contemporary data demonstrate that ICSI significantly improves fertilization rates and live birth rates (LBR) in high-severity cases by directly circumventing physiological barriers to natural conception [2]. Reported fertilization rates consistently range between 50% and 80%, even in the presence of pronounced sperm morphological abnormalities or severe quantitative deficiencies. This reproducible performance supports ICSI’s position as the definitive therapeutic modality across a broad spectrum of male-derived subfertility etiologies [2, 3].

Although fertilization success rates seem high in ICSI, 1–4% total fertilization failure (TFF) and 15–16% embryo development arrest (EDA) occur in all ICSI cases [4, 5]. Therefore, it is essential to identify the causes of TFF and EDA, the etiology of which has not yet been clarified, to reduce stressful consequences for both the patient and the clinician and maximize the success rate per cycle.

Total fertilization failure (TFF) refers to the fertilization failure of all mature oocytes after oocyte retrieval. Failure rates increase in recurrent ICSI cycles and rise to 13% in second ICSI trials [4]. Factors associated with TFF are generally described as male infertility with impaired semen parameters and sperm morphologies and cycles with low oocyte count and quality [6, 7].

Embryo Development Arrest (EDA) is defined as the complete cessation of embryonic progression where no further cell division or developmental transition (such as compaction or cavitation) is observed over a period of at least 24 h. In the context of the ESHRE/ALPHA consensus, this is typically characterized by the failure of the embryo to progress to the next developmental stage within the expected timeframe, confirmed by stagnant morphological or morphokinetic parameters in two consecutive observations 24 h apart [7, 8].

The primary aim of this study is to identify factors associated with total fertilization failure and embryo development arrest.

## Materials and methods

Data of patients admitted to the Reproductive Assisted Therapy Clinic of Ankara Etlik Zübeyde Hanım Gynecology Training and Research Hospital for infertility treatment were retrospectively analyzed. A total of 2,039 ICSI cycles performed between January 2016 and December 2019 were assessed for eligibility. After exclusion of 193 cycles (uncontrolled systemic disease, *n* = 32; insufficient data, *n* = 41; no oocyte retrieval, *n* = 67; no viable sperm, *n* = 53), 1,846 eligible cycles were included in the final analysis.

**Fig. 1:**
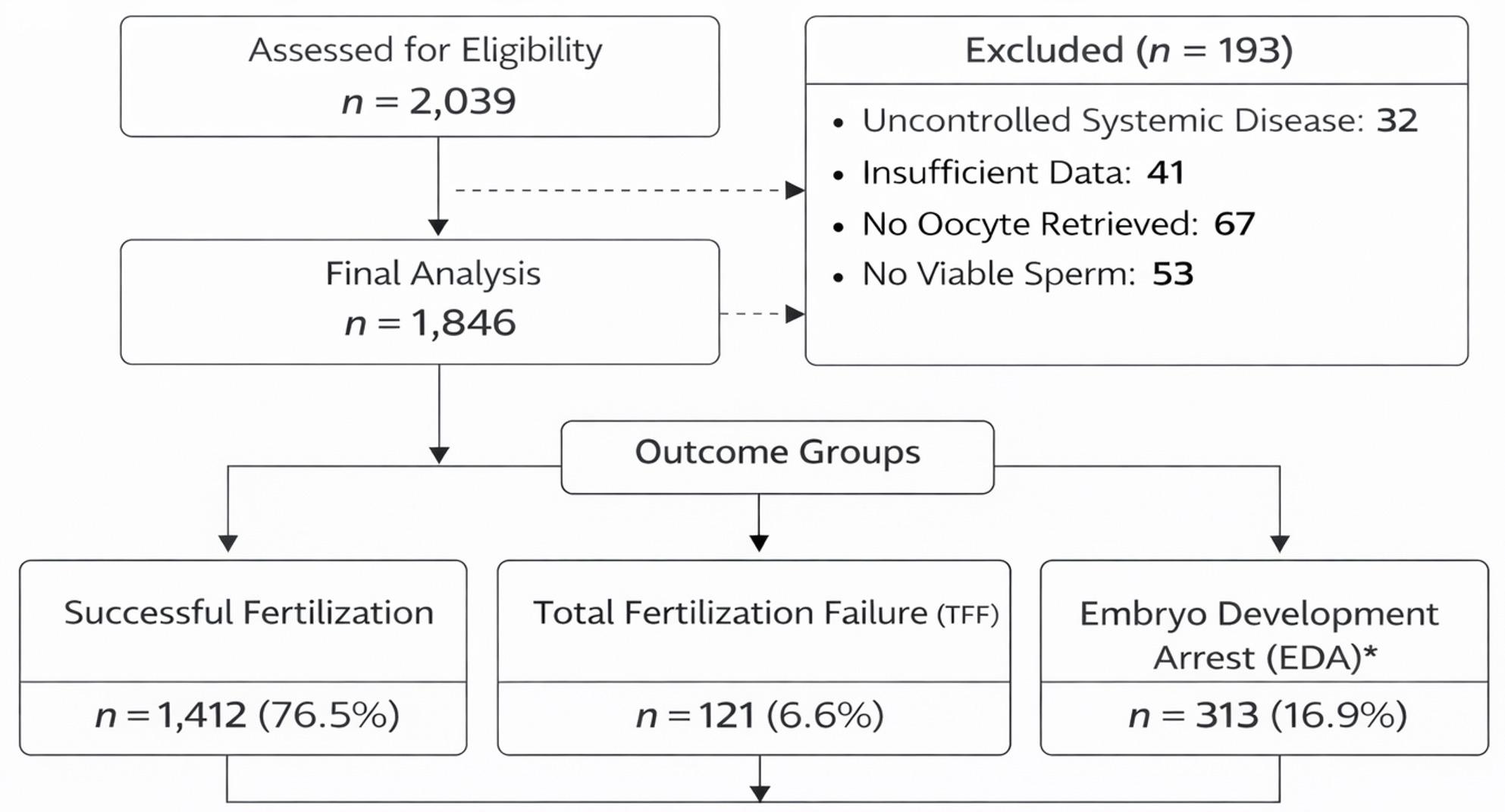
Study flowchart

The final cohort was allocated into three outcome groups: Successful Fertilization (*n* = 1,412; 76.5%), Total Fertilization Failure (TFF; *n* = 121; 6.6%), and Embryo Development Arrest (EDA; *n* = 313; 16.9%). Patient records were retrieved from the institutional clinical database following approval by the university ethics committee. The study flowchart illustrating cycle screening and final allocation is presented in Fig. 1.

Baseline clinical and ovarian reserve parameters included female age, body mass index (BMI), basal follicle-stimulating hormone (FSH), basal estradiol level, serum anti-Müllerian hormone (AMH) level, antral follicle count (AFC), duration of infertility, infertility etiology, male age, sperm retrieval method, and semen analysis parameters. Baseline variables were defined as characteristics available prior to oocyte retrieval and fertilization assessment.

Embryological outcome parameters comprised the number of retrieved oocytes, the number of metaphase II (MII) oocytes, and fertilization status assessed 16–18 h after ICSI. Fertilization was confirmed by the presence of two distinct pronuclei (2PN) and two polar bodies. Cycles in which no oocytes exhibited 2PN formation were classified as total fertilization failure (TFF). Embryo culture was performed using a sequential medium system with daily morphological assessment in accordance with established embryo evaluation guidelines [4, 9, 10].

EDA was defined according to the 2025 ESHRE consensus as complete cessation of embryonic cleavage for a minimum of 24 h, confirmed by absence of developmental progression in two consecutive observations despite optimal culture conditions. Embryos meeting these criteria were classified in the EDA group.

For comparative analyses, the successful fertilization group (Group I, *n* = 1412) and the unsuccessful fertilization group comprising TFF and EDA cycles (Group II, *n* = 434) were evaluated.

To avoid overadjustment and collider bias, post–oocyte retrieval and post-insemination variables, including retrieved oocyte number, MII oocyte count, OPU-day estradiol level, total gonadotropin dose, and 2PN count were excluded from multivariable modeling. The final multivariable logistic regression model was restricted to baseline pre-treatment variables available prior to oocyte retrieval and ICSI, thereby ensuring temporal consistency between predictors and outcome.

Serum AMH levels were log-transformed due to right-skewed distribution. Multicollinearity was assessed using variance inflation factor (VIF) analysis, and all covariates demonstrated VIF values below 3, indicating absence of significant collinearity. Model calibration was evaluated using the Hosmer–Lemeshow goodness-of-fit test. Discriminative performance was assessed by calculating the area under the receiver operating characteristic curve (AUC) based on predicted probabilities derived from the final multivariable model.

Internal validation of the final multivariable logistic regression model was performed using bootstrap resampling with 1,000 iterations. In each bootstrap sample, the model was refitted and its discriminative performance was evaluated. Optimism was estimated as the mean difference between bootstrap AUC and test AUC, and an optimism-corrected AUC was calculated to provide a more reliable estimate of model discrimination adjusted for potential overfitting.

### Data analysis and statistics

All statistical analyses were performed using IBM SPSS Statistics version 22.0 (22.0, SPSS Inc., Chicago, IL, USA). The distribution of continuous data was determined using the Kolmogorov-Smirnov test of normality. Since continuous data did not show normal distribution, median, minimum, and maximum values were calculated with descriptive statistics. Mann-Whitney U test was used to compare two groups of continuous data showing non-parametric distribution, and the Kruskal Wallis test was used to compare the three groups. Chi-square and Fisher exact test were used for categorical variables between groups. Bonferroni correction and Mann-Whitney U test were used for pairwise comparisons of data in which significant differences were detected in triple comparisons. Spearman correlation analysis was used for correlations between variables. To evaluate factors associated with fertilization success, univariate logistic regression analyses were initially performed. Variables demonstrating statistical significance in univariate analysis (*p* < 0.10) and those considered clinically relevant were subsequently entered into a multivariable logistic regression model to estimate adjusted odds ratios (ORs) with 95% confidence intervals (CIs). Model assumptions were evaluated prior to analysis.

All statistical tests were two-tailed, and a p-value < 0.05 was considered statistically significant.

## Results

The successful fertilization rate, defined as cycles yielding transferable-quality embryos, was 76.5% (1412/1846). Total fertilization failure (TFF) occurred in 6.6% (121/1846) of cycles, while embryo developmental arrest (EDA) was observed in 16.9% (313/1846).

Fertilization success varied significantly according to infertility etiology (*p* < 0.001, Table 1). The highest success rates were observed in ovulatory dysfunction (97.5%) and male factor infertility (92.6%), followed by unexplained infertility (85%). In contrast, lower success rates were observed in diminished ovarian reserve (59.2%), endometriosis (32.1%), and tubal factor infertility (35.3%).

**Table 1:** Comparison of cases according to fertilization outcome (TFF, EDA, and successful fertilization)

	Successful Fertilization (Group 1) *n*:1412	Embryo Development Arrest and Total Fertilization Failure (Group 2) *N*:434	*p*
Unexplained infertility	85% (198/231)	15% (33/231)	< 0.001
Male factor	92.6% (416/449)	7.4% (33/449)	
DOR	59.2% (134/226)	40.8% (92/226)	
Endometriosis	32.1% (45/140)	67.9% (95/140)	
Ovulatory Dysfunction	97.5% (527/540)	2.5% (13/540)	
Tubal factor	35.3% (92/260)	64.7% (168/260)	
Total	1412	434	

*DOR* Diminished ovarian reserve

When successful and unsuccessful cycles (TFF + EDA) were compared (Table 2), no statistically significant differences were observed in BMI, basal estradiol level (day 3), ovulation induction duration, or infertility duration (*p* > 0.05). However, female age and basal FSH levels were significantly lower in the successful fertilization group (median age: 31 vs. 33 years, *p* < 0.001; median basal FSH: 7.69 vs. 9.2 mIU/mL, *p* < 0.001).

**Table 2:** Comparison of cases according to fertilization success

	Successful Fertilization (Group 1)	Embryo Development Arrest and Total Fertilization Failure (Group 2)	*p*
	*n*:1412	*N*:434	
Female Age, year	31 (19–46)	33 (18–47)	< 0.001
BMI, kg/m^2^	25.6 (15.7–44.8)	26 (17-45.4)	0.265
FSH, mIU/mL	7.69 (0.48-62)	9.2 (0.71-42)	< 0.001
Estradiol, pg/mL	47.9 (11.8–482)	44 (11.6–596)	0.874
OPU day estradiol level, pg/mL	1310 (97-7731)	785 (58-5959)	< 0.001
AMH, ng/mL	1.81 (0.01-33)	0.7 (0.01-74)	< 0.001
Antral Follicle Count	10 (0–30)	6 (0–30)	< 0.001
Administered gonadotropin total dose	2025 (675–7800)	2400 (688–5550)	< 0.001
Ovulation induction time, day	10 (6–17)	10 (5–15)	0.062
OPU day collected oocyte count	10 (1-144)	5 (1–31)	< 0.001
Obtained M2 oocyte count by OPU	8 (1–32)	3 (0–26)	< 0.001
Infertility period, month	54 (1-288)	57 (2-276)	0.642
2PN count	4 (1–28)	1 (0–16)	< 0.001

*BMI* body mass index, *FSH* follicle-stimulating hormone, *OPU* oocyte pick up, *AMH* anti-müllerian hormone

Serum AMH levels and antral follicle count were significantly higher in cycles with successful fertilization (AMH: 1.81 vs. 0.7 ng/mL; AFC: 10 vs. 6; both *p* < 0.001). As expected, OPU-day estradiol levels and the number of retrieved and MII oocytes were also higher in successful cycles (*p* < 0.001), reflecting improved ovarian response.

Analysis according to sperm retrieval technique showed that the majority of sperm samples were obtained from ejaculate (1604/1746), followed by TESE (132/1746). Fertilization success rates were 75.4% for ejaculate samples and 70.4% for TESE, with no statistically significant difference among retrieval methods (*p* = 0.771). Similarly, total sperm count, motility, progressive motile sperm count, and Kruger morphology were not significantly associated with fertilization outcome (*p* > 0.05). Although male age differed slightly between groups (33 vs. 34 years, *p* < 0.01), this difference did not persist in multivariable analysis.

When the three outcome groups (TFF, EDA, and successful fertilization) were analyzed separately (Table 3), a stepwise gradient was observed for female age, ovarian reserve markers, and ovarian response variables. Female age increased progressively from successful fertilization to EDA and TFF (31 vs. 33 vs. 35 years, *p* < 0.001). Similarly, AMH and AFC were lowest in TFF, intermediate in EDA, and highest in successful cycles (*p* < 0.001). Basal FSH showed the opposite pattern.

**Table 3:** Comparison of cases according to fertilization outcome (TFF, EDA, and successful fertilization)

	TFF *n*= 121	EDA *n*= 313	Successful Fertilization *n*= 1412	***p***
Female Age, year	35 (24–47)^a^	33 (18–46)^b^	31 (19–46)^c^	< 0.001
BMI, kg/m^2^	26.9 (17–45)	25.75 (17–43)	25.8 (15.7–44.8)	0.153
FSH, mIU/mL	10.7 (2.48–22.92)^a^	8.9 (0.7–42)^a^	7.7 (0.42-62)^b^	< 0.001
Estradiol, pg/mL	43 (11.8–178)	45.1 (15–496)	47.5 (11.8–492)	0.983
OPU day estradiol level, pg/mL	589.8 (69.1-5256.5)^a^	821.5 (58.3-5852.9)^b^	1310.3 (97.3-7731.9)^c^	< 0.001
AMH, ng/mL	0.63 (0.1–13)^a^	0.7 (0.1–74)^a^	1.75 (0.1–33) ^b^	< 0.001
Antral Follicle Count	5 (0–30)^a^	7 (0–30)^b^	10 (0–30)^c^	< 0.001
Administered gonadotropin total dose	2400 (1100–5250)^a^	2250 (688–5550)^a^	2025 (800–5250)^b^	< 0.001
OPU day collected oocyte count	4 (1–29)^a^	5 (1–31)^b^	10 (1–43)^c^	< 0.001
Obtained M2 oocyte count by OPU	2 (0–20)^a^	4 (0–26)^b^	8 (1–32)^c^	< 0.001
Infertility period, month	48 (7-228)	60 (2-276)	48 (1-264)	0.881
2PN count	-	1 (0–16)^a^	4 (0–28)^b^	< 0.001

*BMI* body mass index, *FSH* follicle stimulating hormone, *OPU* oocyte pick up, *AMH* anti-müllerian hormone

a, b, c: groups with different superscript letters differ significantly (Bonferroni-adjusted pairwise comparison, *p* < 0.05)

In contrast, BMI, basal estradiol level, ovulation induction duration, and infertility duration did not differ significantly among the three groups.

In the multivariable logistic regression model restricted to baseline pre-treatment variables (Table 4), female age, basal FSH level, serum AMH level (log-transformed), and infertility etiology remained independently associated with fertilization success. Increasing female age and higher basal FSH levels were associated with decreased odds of successful fertilization, whereas higher serum AMH levels were positively associated with fertilization success.

**Table 4:** Univariate and multivariate logistic regression analysis of factors predicting fertilization success

Variable	*p*-value	Adjusted OR (95% CI)	*p*-value
Female age (year)	< 0.001	0.94 (0.91–0.97)	< 0.001
BMI (kg/m²)	0.265	0.99 (0.96–1.02)	0.580
Infertility duration (month)	0.642	1.00 (0.99–1.01)	0.720
Basal FSH (mIU/mL)	< 0.001	0.93 (0.89–0.97)	0.002
Basal estradiol (pg/mL)	0.874	1.00 (0.99–1.01)	0.910
Serum AMH (log-transformed)	< 0.001	1.42 (1.18–1.72)	< 0.001
Male age (year)	< 0.01	0.98 (0.95–1.01)	0.180
Total progressive motile sperm count	> 0.05	1.00 (0.99–1.01)	0.470
Kruger morphology (%)	> 0.05	1.01 (0.98–1.03)	0.310
Sperm retrieval method (TESE vs. ejaculate)	0.771	0.91 (0.61–1.37)	0.650

*BMI* body mass index, *FSH* follicle stimulating hormone, *OPU* oocyte pick up, *AMH* anti-müllerian hormone

Male age, semen parameters, and sperm retrieval method were not independently associated with fertilization outcome after adjustment. Multicollinearity diagnostics demonstrated VIF values < 3 for all covariates, confirming model stability. The apparent discriminative performance of the final multivariable model yielded an AUC of 0.64. Following bootstrap internal validation, the optimism-corrected AUC was 0.63, indicating minimal overfitting and stable model performance. Although statistically significant associations were observed for several baseline predictors, the overall discriminative ability of the model was modest.

## Discussion

Fertilization failure and embryo developmental arrest remain clinically significant and psychologically distressing outcomes in ICSI cycles. Despite continuous advances in assisted reproductive technologies, adverse outcomes persist. Reported total fertilization failure (TFF) rates range between 3% and 6%, while early embryo developmental arrest occurs in approximately 15–16% of cycles [5, 6, 11, 12]. In our cohort, TFF (6.5%) and EDA (16.9%) were consistent with these published data.

Fertilization failure following ICSI is increasingly recognized as multifactorial. Oocyte activation deficiency, molecular gamete defects, and chromosomal abnormalities represent key biological mechanisms [13, 14]. Concurrently, evolving guidelines in ovarian stimulation, laboratory practice, and embryo assessment emphasize the need to interpret clinical outcomes within contemporary embryological standards [8, 10, 15].

Historically, severe male factor infertility was considered a primary contributor to TFF. However, accumulating evidence suggests that ICSI largely mitigates the impact of conventional semen abnormalities. Consistent with prior reports [4, 6, 7, 16], we found no independent association between semen parameters or sperm retrieval method and fertilization outcomes after multivariable adjustment. This finding reinforces the concept that, once ICSI is employed, oocyte-related determinants play a dominant role in early developmental competence.

In contrast, ovarian reserve markers demonstrated consistent associations with fertilization outcomes. Unsuccessful cycles were characterized by higher female age, elevated basal FSH levels, and lower AMH and AFC values. Importantly, when multivariable modeling was restricted to baseline pre-treatment variables, female age, basal FSH, serum AMH, and infertility etiology remained independently associated with fertilization success. This approach ensured temporal alignment between predictors and outcome and avoided incorporation of post-retrieval intermediates into the regression model.

The attenuation of age effects after adjustment for ovarian reserve markers suggests partial mediation through declining ovarian reserve rather than a purely chronological effect. This interpretation is consistent with current evidence linking age-related meiotic errors, aneuploidy, mitochondrial dysfunction, and impaired cytoplasmic maturation to reduced oocyte competence [5, 14, 17]. Thus, ovarian reserve parameters may function as clinically measurable proxies for underlying biological aging processes within the oocyte.

Although ovarian response variables (e.g., retrieved oocyte number and MII count) differed across outcome groups in univariate analyses, these factors lie downstream of baseline ovarian reserve and were therefore excluded from multivariable modeling to prevent overadjustment bias. By restricting analysis to pre-treatment characteristics, the final model provides clinically actionable risk estimation prior to cycle initiation rather than post hoc confirmation.

The model demonstrated modest discriminative performance, reflecting the inherently multifactorial nature of fertilization competence. Oocyte maturation dynamics, spindle integrity, mitochondrial function, and molecular activation pathways, none of which were directly assessed in this study likely contribute to residual unexplained variability. Therefore, while diminished ovarian reserve markers significantly increase the probability of TFF and EDA, they do not fully account for all biological determinants of early embryonic development [18, 19].

Recent literature (2022–2026) has highlighted oocyte maturation arrest as a distinct contributor to fertilization failure and early embryonic arrest. Arrest at the germinal vesicle (GV) or metaphase I (MI) stage has been associated with spindle abnormalities, mitochondrial dysfunction, and pathogenic variants affecting meiotic progression and oocyte activation. Even morphologically mature (MII) oocytes may exhibit subclinical cytoplasmic immaturity that compromises embryonic genome activation. These observations support the interpretation that TFF and EDA may represent different clinical manifestations along a shared spectrum of compromised oocyte competence [20, 21].

From a translational standpoint, baseline ovarian reserve markers may help identify patients with relatively increased probability of unsuccessful fertilization and may support individualized counseling, although their standalone predictive accuracy remains limited [22]. In women with diminished ovarian reserve or advanced maternal age particularly those with prior unsuccessful cycles closer laboratory monitoring or consideration of adjunctive approaches may be warranted. However, such strategies require validation in prospective and ideally multicenter studies before routine implementation.

This study has limitations. Its retrospective, single-center design precludes causal inference and limits external generalizability. Residual confounding cannot be excluded despite multivariable adjustment. Molecular assessments of oocyte quality, sperm function, or time-lapse morphokinetic parameters were not available. Future prospective studies integrating clinical, laboratory, and molecular biomarkers are necessary to refine predictive modeling and enhance individualized reproductive strategies.

In conclusion, diminished ovarian reserve markers including advanced female age, lower AMH, and higher basal FSH were independently associated with increased risk of fertilization failure and early embryonic developmental arrest in ICSI cycles. These findings underscore the central role of oocyte competence in determining early ART outcomes and support consideration of baseline ovarian reserve parameters as clinically relevant associative markers, while acknowledging that their discriminative capacity is limited when used in isolation. These findings emphasize the associative role of diminished ovarian reserve markers in fertilization outcomes but highlight the need for integration of molecular and laboratory-level variables to improve predictive accuracy.

## Data Availability

The datasets used and/or analyzed during the current study are available from the corresponding author on reasonable request.

## Abbreviations

ICSI: Intracytoplasmic sperm injection
TFF: Total fertilization failure
EDA: Embryo development arrest
IVF: In vitro fertilization
FSH: Follicle-stimulating hormone
AMH: Anti-Müllerian hormone
OPU: Oocyte pick-up
2PN: Two-pronucleus
BMI: Body mass index
TESE: Testicular sperm extraction
PESA: Percutaneous epididymal sperm aspiration
MESA: Microsurgical epididymal sperm aspiration
